# Comparative Analysis of the Hemodynamic Effects of Remimazolam and Propofol During General Anesthesia: A Retrospective Study

**DOI:** 10.7759/cureus.58340

**Published:** 2024-04-15

**Authors:** Shota Tsukimoto, Atsuhiro Kitaura, Rina Yamamoto, Chikara Hirase, Shinichi Nakao, Yasufumi Nakajima, Takuro Sanuki

**Affiliations:** 1 Department of Dental Anesthesiology, Kanagawa Dental University, Yokosuka, JPN; 2 Department of Anesthesiology, Kindai University, Osaka, JPN; 3 Clinical Research Center, Kindai University, Osaka, JPN; 4 Perioperative Management Center, Okanami General Hospital, Mie, JPN

**Keywords:** estimated continuous cardiac output (escco) system, hemodynamic effects, autonomic nerve system, heart rate variability analysis, remimazolam

## Abstract

Purpose: Hypotension is common during anesthesia induction. However, minimal hemodynamic effects of remimazolam anesthesia have been reported. We hypothesized that remimazolam would have weaker hemodynamic effects than would propofol. To test this, we simultaneously evaluated the hemodynamics using the estimated continuous cardiac output (esCCO) system and heart rate variability (HRV) during anesthesia induction.

Methods: This was a single-center, observational, retrospective study of patients undergoing dental surgery under general anesthesia between 2020 and 2022. Seventy patients were divided into two groups: remimazolam (R group; n=34) and propofol (P group; n=36). The information obtained from the anesthesia records, patient information, esCCO system parameters, and HRV were integrated and analyzed. The percentages of various parameters were set to 100% for the pre-induction phase as the baseline.

Results: The %MAP (noninvasive mean arterial blood pressure) decreased over a narrower range in the R compared to the P group (-17.8% (-26.3%, -11.9%) vs. -22.6% (-32.9%, -17.0%); P=0.039). The %HR (heart rate) increased significantly in the R group and decreased in the P group (+10.7% (+6.5%, +18.6%) vs. -6.5% (-14.5%, +8.4%); P<0.01). The %SV_esCCO_ (stroke volume calculated using the esCCO system) decreased significantly in both groups, but the R group showed a smaller difference compared to the P group (- 5.1% (-7.7%, -2.1%) vs. -10.0% (-13.8%, -5.6%); P<0.01). The rates of change in %LF nu (normalized unit of low frequency) and %HF nu (normalized unit of high frequency) were lower for the R than for the P group, although the difference was not significant (+6.8% (-14.5%, 32.4%) vs. +9.2% (-7.2%, +59.7%), P=0.30; +7.9% (-51.0%, +66.9%) vs. +22.8% (-26.1%, +61.6%), P=0.57).

Conclusion: Remimazolam demonstrated a lower MAP reduction rate than propofol. A compensatory increase in HR occurred with a decrease in stroke volume. However, the HR increase was not attributable to the autonomic nervous system.

## Introduction

Hypotension is one of the most common immediate adverse events after anesthesia induction, affecting up to 99% of patients undergoing surgery. Hypotension can also have a significant impact on perioperative organ ring dysfunction and postoperative outcomes; therefore, perioperative hemodynamic stability is important [1-3].

Remimazolam is a relatively new benzodiazepine anesthetic with a short duration of action, low hemodynamic effects, availability of a reliable antagonist, absence of vascular pain during anesthesia induction, and the possibility of continuous induction of anesthetic drugs. Furthermore, it has a shorter half-life than other benzodiazepines because it is hydrolyzed by carboxylesterases [4-8].

Several studies have been conducted on patients undergoing general anesthesia with remimazolam, assessing heart rate variability (HRV) [9] and hemodynamics in a noninvasive manner [10,11]. However, there are no reports on the simultaneous use of these two methods to examine hemodynamics. By analyzing the results obtained in our study, we believe that we may be one step closer to elucidating the mechanism by which the induction of anesthesia with remimazolam is less likely to cause hypotension.

To elucidate this mechanism, we simultaneously evaluated the hemodynamics using the estimated continuous cardiac output system (the esCCO system) and HRV during anesthesia induction, when the blood concentration of anesthetic drugs is the highest, and compared the effects of different anesthetic agents.

## Materials and methods

Study design and patients

**Figure 1:**
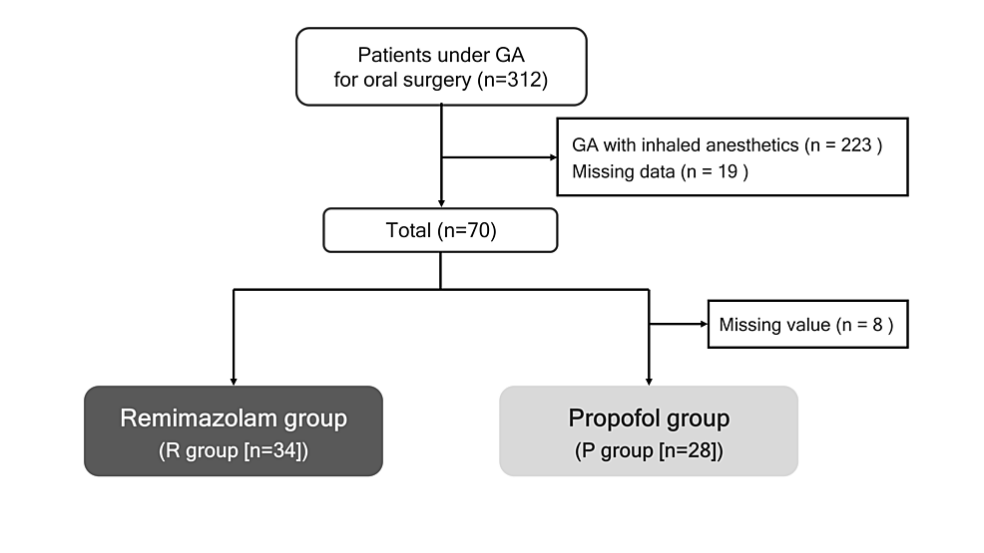
Flowchart of the study. Of the 312 patients who underwent dental surgery with GA, 242 were excluded and 70 were included in the analysis. Of the 70 patients included, 34 were included in the R group and 36 were included in the P group. Of the 36 patients in the P group, 8 were excluded from the analysis as they had missing data. GA: general anesthesia

This retrospective observational study was approved by the Ethics Committee of Kindai University Faculty of Medicine (approval no. R04-029), according to the principles of the Declaration of Helsinki. We have registered this clinical study with the University Hospital Medical Information Network (UMIN) (https://center6.umin.ac.jp/cgi-bin/ctr_e/ctr_view.cgi?recptno=R000050614 (UMIN000045998; principal investigator: Shota Tsukimoto, registered on November 21, 2021)). The opt-out method was used to obtain consent in this study, and the opt-out notice is available at https://www.med.kindai.ac.jp/anes/clinical_study.html. This study included patients who underwent dental surgery under general anesthesia (GA) at Kindai University Hospital between April 2020 and May 2022. In total, 312 patients were included in this study (Figure 1).

Anesthetic methods

After entering the operating room, vital sign monitors for electrocardiography, pulse oximetry, noninvasive blood pressure (MAP), and bispectral index (BIS) (Aspect Medical Systems, USA) were attached to the patient. Cardiac output (CO), stroke volume (SV), and cardiac index (CI), which can only be measured invasively, were calculated noninvasively and continuously using an electrocardiogram (ECG) and the pulse waves obtained from the pulse oximeter (esCCO system; Nihon Kohden Tokyo, Japan). The pre-induction stage was defined as the period during which all patients had BIS values ≥90 and a Modified Observer’s Assessment of Alertness/Sedation (MOAA/S) scale score of 3 or higher. The post-induction stage was the period during which all patients had BIS values ranging from 40 to 60 and MOAA/S scale scores <1 after anesthetic induction.

**Figure 2:**
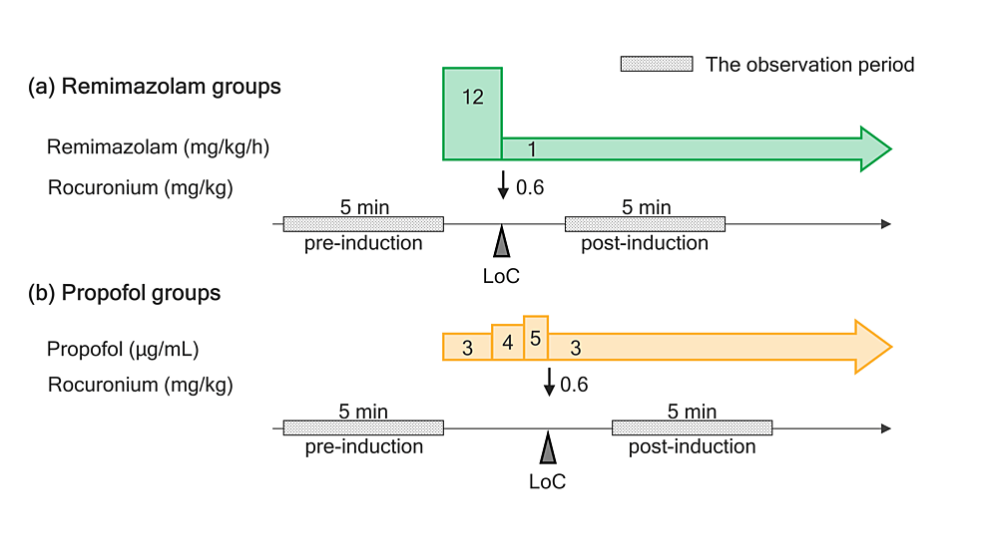
Anesthetic methods of the study. (a) The anesthetic method of the remimazolam group, (b) the anesthetic method of the propofol group. LoC was determined using BIS values and MOAA/S scale scores. In the post-induction stage, we pick up cases without narcotic analgesics in which only intravenous anesthetics and muscle relaxants are administered. LoC: loss of consciousness; BIS: bispectral index; MOAA/S: modified observer’s assessment of alertness/sedation

Anesthesia was induced in both groups after securing intravenous access. Remimazolam anesthesia was induced at 12 mg/kg/h and decreased to 1 mg/kg/h after loss of consciousness (LoC) in the remimazolam group (Group R). Propofol was administered using a target-controlled infusion (TCI) system (TE-SS830T, Terumo Medical Corp.). The first effect-site target propofol concentration was 3 μg/mL. If the MOSS/A scale score did not fall below 1 or the BIS value did not fall below 60, the effect-site target blood concentration was increased by 1 μg/mL every 2 min. After LoC, the effect-site target propofol concentration returned to 3 μg/mL (Group P). Thereafter, the BIS value was maintained at 40-60 during anesthesia and the dosage was adjusted at the discretion of the anesthetist. After muscle relaxants were administered and the response was observed, noninvasive positive pressure ventilation with a mask was performed without intubation under the following conditions: no positive end-expiratory pressure (PEEP), respiratory rate of 10 breaths/min, end-tidal carbon dioxide tension (EtCO_2_) maintained at 35-45 mmHg, and positive pressure not exceeding 20 cmH_2_O. The anesthesia method is illustrated in Figure 2.

Outcomes

The primary outcome was the change in the percentage of noninvasive mean arterial blood pressure (%MAP) from the pre- to post-induction phases for each anesthetic agent. MAP was measured with a cuff on the upper arm every 2.5 minutes.

The secondary outcomes were the data from the anesthesia record (duration of anesthesia, duration of surgery, ECG findings, heart rate (HR), and BIS value), patient information (age, sex, body mass index, classification of American Society of Anesthesiologists physical status (ASA-PS) and history of hypertension), parameters of the esCCO system (cardiac output (CO_esCCO_), stroke volume (SV_esCCO_), and cardiac index (CI_esCCO_) calculated in this system during the pre- and post-induction periods), and the HRV from the RR interval of the ECG. The percentages of various parameters were set to 100% for the pre-induction phase as the baseline. These data were obtained from electronic medical records.

Analysis of HRV

To investigate the influence of anesthetic agents on the autonomic nervous system (ANS), we used the analysis of HRV during the induction of anesthesia, which is a noninvasive and conventional method in many research fields [12-21]. The methodology for analyzing the HRV was adapted from previous studies [9,15]. In this study, we used MemCalc/Tonam2 (Suwa Trust, Tokyo, Japan) to analyze the HRV from ECGs. Total power (0-0.4 Hz, ms^2^), low frequency power (LF; 0.04-0.15 Hz, ms^2^), and high frequency power (HF; 0.15-0.4 Hz, ms^2^) were determined using power spectrum density analysis of the R-R interval (RRI) variability. Normalized units of LF (LF nu) and HF (HF nu) were calculated to assess the balance between sympathetic and parasympathetic activity [15]. Nu (%) was calculated as follows: LF nu (HF nu) = 100 × LF (HF)/(LF + HF).

Statistical analysis

All outcomes were compared between the R and P groups using the Mann-Whitney U test. The pre- and post-induction changes in the same anesthetic group were tested using the Wilcoxon signed-rank test. Data were expressed as the median and interquartile range (IQR), and statistical significance was set at P<0.05. All statistical analyses were performed using GraphPad Prism 9.5.1 (Dotmatics, USA).

## Results

Patient background

Of the 312 patients who underwent dental surgery with GA, 242 were excluded and 70 were included in the analysis. Of the 70 patients included, 34 were included in the R group and 36 were included in the P group. Of the 36 patients in the P group, eight were excluded from the analysis as they had missing data (Figure 1).

As shown in Table 1, there were no significant differences in sex, age, body mass index, or ASA-PS scores. In addition, the time to LoC was significantly shorter in the R group than in the P group. Furthermore, there was no significant difference in the number of patients with hypertension between the groups.

**Table 1:** Demographic data of the two anesthetic groups. There were no significant differences in sex, age, body mass index, or ASA-PS scores. In addition, the time to LoC was significantly shorter in the R group than in the P group. Furthermore, there was no significant difference in the number of patients with hypertension between the groups. Values are presented as n, (%), or median (IQR). Statistical methods for P-values: (a) chi-squared test and (b) Mann–Whitney U test. ASA-PS: classification of American Society of Anesthesiologists physical status; LoC: loss of consciousness; IQR: interquartile range

	Remimazolam Groups (n=34)	Propofol Groups (n=28)	P-value
Sex (Male/Female)	15 (44.1)/19 (55.9)	16 (57.1)/12 (42.9)	0.44^a^
Age	42 (27.8, 72.3)	60 (27.5, 70.8)	0.50^b^
Body mass index	21.7 (20.4, 24.2)	23.1 (21.4, 25.8)	0.06^b^
ASA-PS (1/2)	13 (38.2)/21 (61.8)	7 (25.0)/21 (75.0)	0.29^a^
Duration of surgery (min)	137.0 (115.8, 161.8)	146.0 (120.0, 181.0)	0.52^b^
Duration of anesthesia (min)	53.5 (37.0, 82.0)	62.0 (25.5, 102.0)	0.93^b^
Time to LoC (sec)	112.5 (92.5, 129.0)	147.0 (112.3, 192.3)	<0.001^b^
History of hypertension	7 (20.6)	7 (25.0)	0.77^a^

Changes in hemodynamic parameters during pre- and post-induction

**Figure 3:**
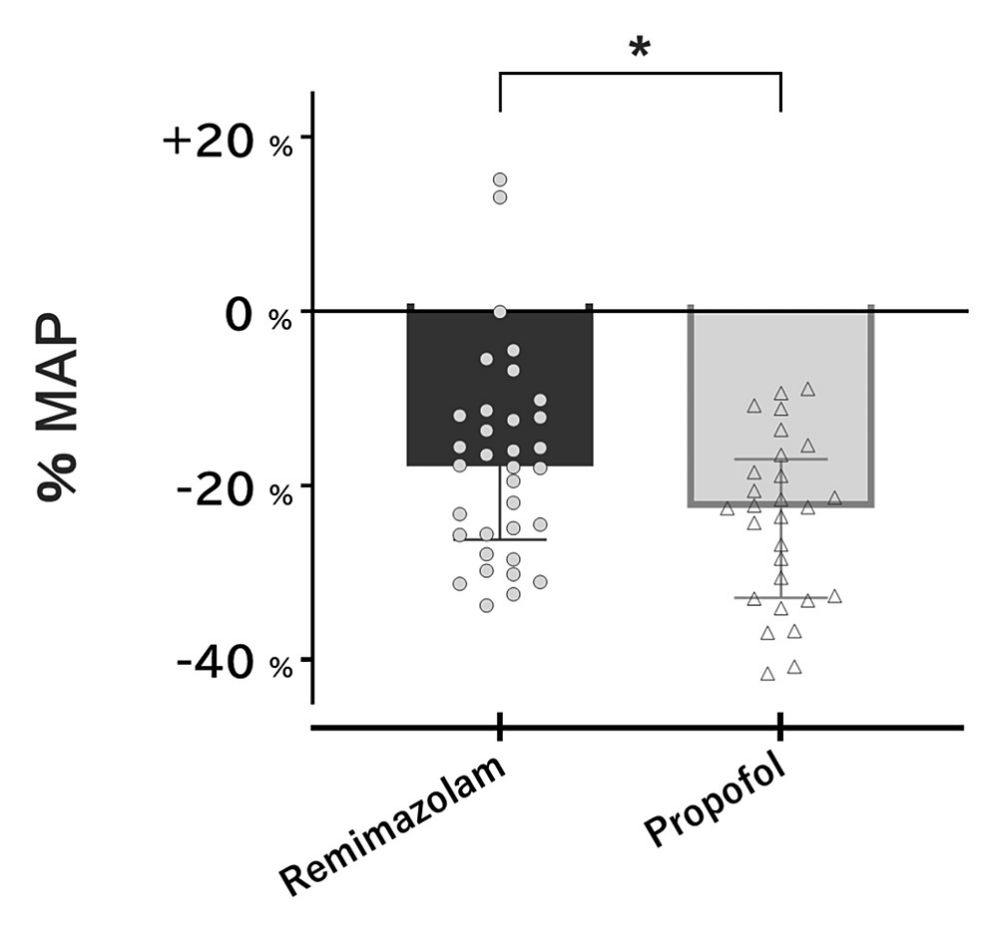
Changes in the percentage of MAP. The %MAP during anesthesia induction with remimazolam and propofol is demonstrated. The change in each study participant is represented by a gray circle (R group) or white triangle (P group). * P < 0.05 (Mann–Whitney U) and versus the R group. MAP: noninvasive mean arterial blood pressure

The changes in the percentage of MAP (%MAP) are shown in Figure 3. The %MAP showed a significant decrease in both groups, with a significant difference in the rate of change between the groups (-17.8% (-26.3%, -11.9%) vs -22.6% (-32.9%, -17.0%); P=0.04).

**Figure 4:**
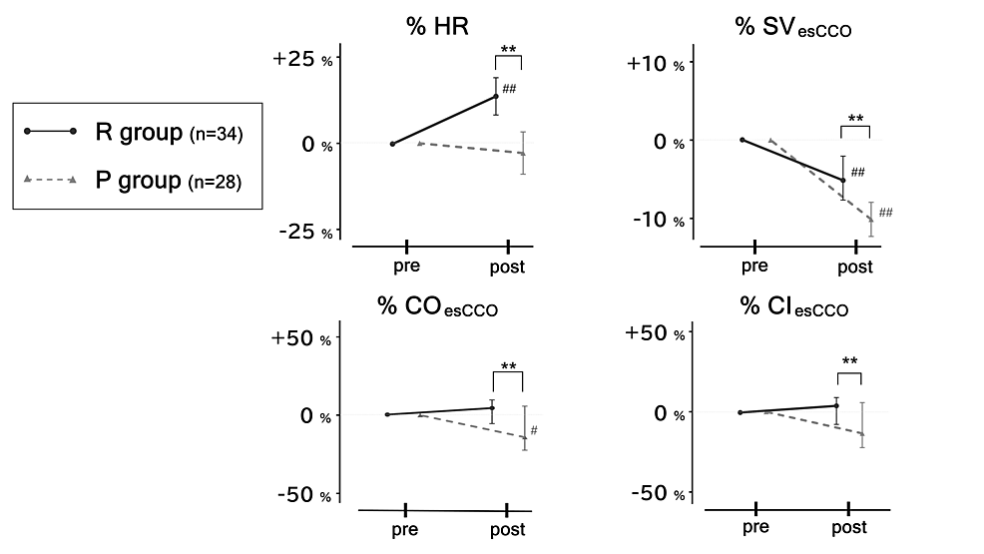
Changes in the noninvasive cardiovascular parameters. The rates of change in HR and the three parameters from the pre- to post-induction phases are shown. The black circles and solid black lines indicate R-group changes, whereas the gray triangles and gray dashed lines indicate P-group changes. *P<0.05 and **P<0.01 vs. the R group. #P<0.05, ##P<0.01, vs. the post-induction phase in each group. HR: heart rate; SV_esCCO_: estimated continuous stroke volume calculated by the esCCO system; CO_esCCO_: estimated continuous cardiac output calculated by the esCCO system; CI_esCCO_: estimated continuous cardiac index calculated by the esCCO system

**Figure 5:**
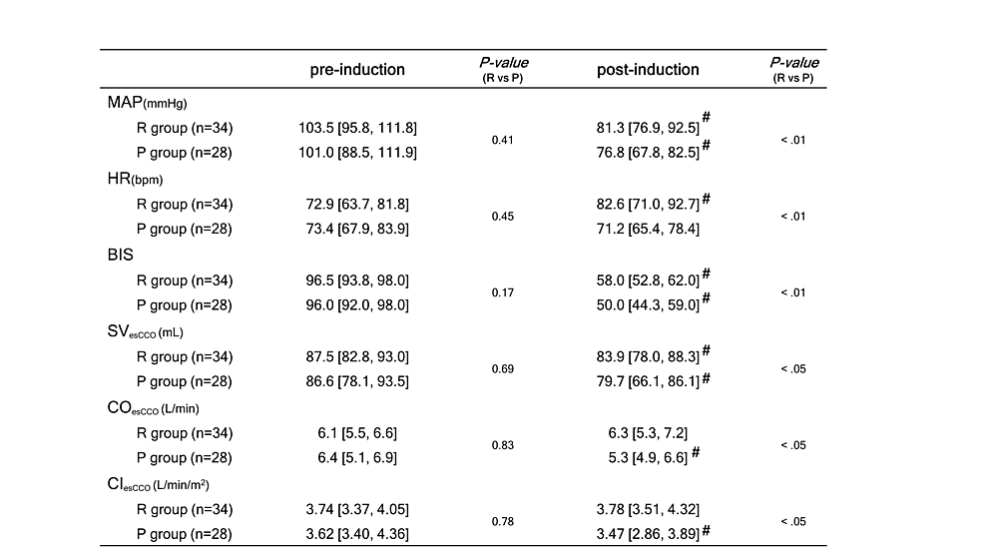
Measurement of noninvasive cardiovascular parameters pre- and post-induction. Measurements of various parameters are expressed as medians with interquartile ranges (IQR). MAP: noninvasive mean arterial blood pressure; HR: heart rate; BIS: bispectral index; SV_esCCO_: estimated continuous stroke volume calculated by the esCCO system; CO_esCCO_: estimated continuous cardiac output calculated by the esCCO system; CI_esCCO_: estimated continuous cardiac index calculated by the esCCO system

Figure 4 shows the %HR and parameters calculated using the esCCO system (%SVesCCO, %COesCCO, and %CIesCCO) for each group. The %HR increased significantly in the R group and decreased in the P group. There was a significant difference in the rate of change between the groups (+10.7% (+6.5%, +18.6%) vs. -6.5% (-14.5%, +8.4%); P<0.01). The %SVesCCO decreased significantly in both groups, but the magnitude of the change was lower in the R group than in the P group (- 5.1% (-7.7%, -2.1%) vs. -10.0% (-13.8%, -5.6%); P<0.01). The %COesCCO values pre- and post-induction increased in the R group and decreased in the P group, with significant differences in the magnitude of change (+4.1% (-5.8%, +9.3%) vs -7.4% (-22.4%, +5.8%); P<0.01). The %CIesCCO showed a trend toward an increase in the R group and a decrease in the P group, but there was also a difference between the magnitudes of change in both groups (+4.1% (-7.3%, +9.3%) vs. -7.4% (-22.3%, +5.8%); P<0.01). Figure 5 denotes the real number of parameters (including BIS) at pre- and post-induction associated with Figures 3-4 in both the R and P groups.

Evaluation of the balance of the ANS via HRV

Analysis of HRV in this study revealed the following three findings.

(1) Pre and post the induction of anesthesia, there was little change in the balance of ANS (%LF nu and %HF nu) in the R group (+6.9% (-14.4%, 31.6%), P=0.08 and +7.9% (-51.0%, +66.9%), P=0.46).

(2) During the same period, the balance of ANS in the P group was significantly tilted toward sympathetic nervous activity (+9.2% (-7.2%, +59.7%), P<0.05 and +22.8% (-26.1%, +61.6%), P<0.05).

(3) There was no statistically significant difference in the changes of %LF and %HF between the two groups (+6.9% (-14.4%, 31.6%) vs. +9.2% (-7.2%, +59.7%), P=0.28; +7.9% (-51.0%, +66.9%) vs. +22.8% (-26.1%, +61.6%), P=0.57).

**Figure 6:**
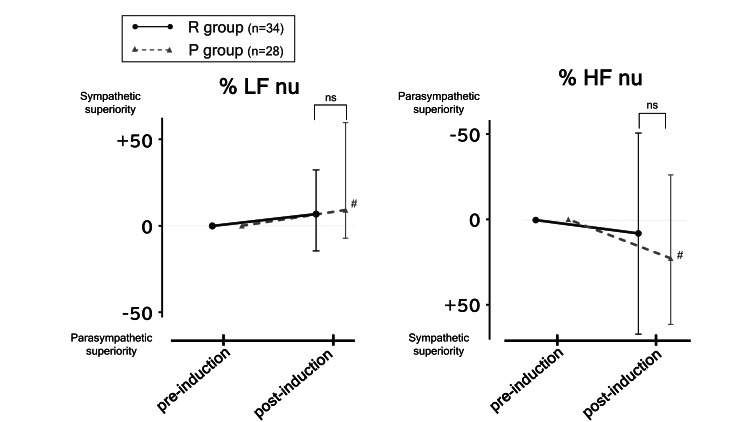
Differential influence of remimazolam and propofol on the balance of the autonomic nervous system (ANS). Although anesthetics were not comparable, it was found that there was a trend toward sympathetic activity in the P group, whereas there was no change in ANS balance in the R group. #P<0.05 vs. the post-induction phase in each group.

In summary, although the anesthetic agents could not be compared, the P group showed a tendency toward sympathetic activity, while there was no alteration in ANS balance in the R group (Figure 6).

## Discussion

We investigated the hemodynamics of remimazolam and propofol administered during anesthesia induction in patients by simultaneously measuring HRV and the esCCO parameters (CO, etc.) using noninvasive methods. There are three major findings of this study. First, remimazolam anesthesia produced a narrower range of MAP reduction than did propofol anesthesia. Second, remimazolam anesthesia decreased SV and increased HR. Moreover, it is unlikely that the increase in HR caused a decrease in SV. Rather, the increase in HR was caused by a compensatory response to the decrease in SV. Third, remimazolam anesthesia did not alter ANS balance. Consequently, we found that remimazolam anesthesia was not associated with significant hemodynamic effects compared to propofol anesthesia.

Perioperative hypotension is also associated with postoperative cardiac injury and other adverse events [22-25]. Hypotension during anesthesia induction is the most common complication during surgery [26,27]. Recent studies have reported that even a brief duration of hypotension during anesthesia induction may lead to postoperative renal injury [27]. Thus, hemodynamic maintenance during anesthesia management is desirable for improving postoperative outcomes.

MAP is the main hemodynamic indicator. Focusing on the difference between MAP before and after the induction of anesthesia, remimazolam anesthesia results in significantly fewer reductions in MAP over a significantly narrower range than propofol anesthesia [9, 28]. The frequency of intraoperative hypotension is 32.1% for remimazolam and 67.9% for propofol [28]. Propofol decreases systemic peripheral vascular resistance, HR, CO, and SV [29]. In contrast, remimazolam decreases MAP by 24%, increases HR by up to 28% [6], and maintains CO better than propofol [28]. No reports exist on the changes in CI during anesthesia induction. This study showed consistent cardiovascular hemodynamics for each anesthetic.

Remimazolam anesthesia has been associated with unexpected increases in HR during GA, presumably from its effects on sympathetic superiority [30]. However, an analysis of HRV before and after the induction of GA showed no change in the ANS with remimazolam [9]. Our results showed the same trend: the balance of the ANS pre- and post-induction of anesthesia was more stable with remimazolam than with propofol. However, there were no significant differences between these anesthetics.

In contrast, propofol is known to (1) suppress the parasympathetic nervous system more strongly than the sympathetic nervous system [12,13] and (2) inhibit the cardiac vagus nerve in the nucleus, which is associated with the parasympathetic nervous system [31].

It is generally believed that an increase in HR is caused by excitation of the sympathetic nervous system; however, this was not the case in the present study. Moreover, from a clinical perspective, we thought it made more sense to consider the increase in HR as a compensatory change due to the decrease in SV. When viewed at in vitro, Urabe et al. found that remimazolam increases intracellular calcium ion concentrations in a volume-dependent manner through calcium mobilization [32]. It has been speculated that remimazolam may influence the uptake of calcium ions by cardiomyocytes, but the mechanism underlying the increase in HR during remimazolam anesthesia is unclear.

Finally, we used the esCCO system, which can measure the estimated continuous CO in a noninvasive manner because the patients in this study underwent a surgical procedure that would cause fewer hemodynamic effects. Our study is similar to that of Sekiguchi et al. [10] in that they investigated the hemodynamic effects of remimazolam using an esCCO system. However, their study differed significantly from ours in that they investigated the effects of remifentanil administration. In other words, no previous study has reported the effects of remimazolam alone.

Remimazolam has been used in several cardiac surgeries owing to its hemodynamic stability [33-35]. It is necessary to consider the potential risks of noncardiac surgery for cardiac disease, as an increase in HR may increase the workload of the heart and lead to changes in the oxygen supply-demand balance of the myocardium, a decrease in coronary perfusion pressure, and the development of myocardial ischemia [36].

This study had several limitations. First, we performed MAP measurements at 2.5-minute intervals. Hemodynamics should be considered in real-time when blood pharmacokinetics change over time, and if we had been able to measure arterial pressure in an invasive method, more detailed data could have been obtained. However, judging from the patient background and the degree of surgical invasion in this study, we think that blood pressure monitoring by an invasive method was unnecessary. Second, CO_esCCO_ shows better trends than arterial pressure-based cardiac output (APCO) and is noninvasive [37]. However, its short-term accuracy, particularly in response to changes in HR, has not yet been established. Third, to accurately assess hemodynamics, peripheral vascular resistance, which can be determined using parameters such as systemic vascular resistance and perfusion index, should be measured. However, this was a retrospective study, and the aforementioned data could not be obtained. Fourth, the timing of the HRV measurement. It is undeniable that patients were in a state of tension immediately after entering the operating room, and their sympathetic activity had already increased and became dominant. However, this should not be a major problem for the pre- and post-induction comparison because the situations with tonic sympathetic activities were the same for both groups. Fifth, HR is easily varied and influenced by many factors, such as the external environment and circadian rhythms [33]. In addition, the amount of data acquired by measurement devices is large, making it easy for the analysis of HRV results to vary. Sixth, propofol anesthesia was delivered using the TCI system, whereas remimazolam was delivered according to the package insert instructions. Therefore, we believe that there was a difference in the time to LoC between the two groups. Seventh, this was a retrospective observational study examining this ratio; however, propensity score matching was not performed.

## Conclusions

The present study concluded that the increase in HR induced by remimazolam anesthesia was not the result of sympathetic activity dominance but was a compensatory change to SV depression. This phenomenon of increased HR may have contributed to the hemodynamic stability. Based on the present results, remimazolam anesthesia may preserve the compensatory responses and physiological reflexes better than propofol anesthesia, and we believe that the compensatory increase in HR due to SV reduction may be one of the reasons for the hemodynamic stability.

Furthermore, by using a combination of the HRV analyzer and esCCO system simultaneously in the same patient, we were able to determine the mechanism by which remimazolam maintained hemodynamic stability.
